# A primer on circadian rhythms for psychiatry

**DOI:** 10.1038/s44277-026-00060-5

**Published:** 2026-05-07

**Authors:** Adriane M. Soehner, Colleen A. McClung

**Affiliations:** 1 Department of Psychiatry, University of Pittsburgh School of Medicine, Pittsburgh, PA USA; 2 Department of Clinical and Translational Science, University of Pittsburgh School of Medicine, Pittsburgh, PA USA

**Keywords:** Circadian rhythms and sleep, Assay systems

Circadian rhythms are fundamental, evolutionary conserved regulators of neural, endocrine, behavioral, and molecular systems that shape diverse aspects of psychiatric function. Disruption to circadian rhythms is increasingly recognized as a key mechanism contributing to the risk, onset, and course of many psychiatric and neurodevelopmental disorders, and represents a scalable target for mechanistic investigation and intervention [1, 2].

## Circadian rhythm regulation

Circadian rhythms are endogenous, approximately 24-hour oscillations in most bodily processes that persist in constant conditions and enable organisms to anticipate daily environmental changes [3]. In humans, the master circadian pacemaker resides in the suprachiasmatic nucleus (SCN) of the anterior hypothalamus. The SCN coordinates most bodily rhythms, including sleep-wake behavior, hormone secretion, metabolism, and affective and cognitive processes. Because the intrinsic period of the SCN varies around 24 h, exogenous time cues (zeitgebers) play a central role in entraining the SCN to the 24-hour day. Environmental light is the dominant zeitgeber. Intrinsically photosensitive retinal ganglion cells (ipRGCs) containing the blue light-sensitive photopigment melanopsin transmit light signals via the retinohypothalamic tract directly to the SCN, synchronizing SCN neurons to the external light-dark cycle. Nonphotic zeitgebers, including physical activity, social interaction, or meals, reach the SCN through indirect pathways. The SCN integrates environmental and behavioral signals to maintain internal temporal alignment with the external day.

At the cellular level, circadian timing is generated by transcription-translation feedback loops, producing ~24 h oscillations in gene expression [2]. These molecular clocks are present in nearly all cells and tissues, forming peripheral oscillators that regulate local transcriptomic and metabolic programs. The SCN synchronizes peripheral clocks through neural and humoral outputs, including melatonin secretion, cortisol rhythms, body temperature cycles, autonomic signals, and behavioral rhythms. This hierarchical organization, in which cell-autonomous molecular clocks are coordinated by the SCN and entrained by environmental zeitgebers, provides a mechanistic framework for understanding how circadian disruption can propagate across brain circuits and peripheral systems implicated in psychiatric illness.

## Evaluating circadian rhythm features

Human circadian rhythms are characterized by three core features – Phase, Period, and Amplitude. **Phase** refers to the position of the internal circadian clock relative to social time. **Period** is largely genetically determined and captures the intrinsic length of the endogenous circadian cycle (tau), typically slightly longer than 24 h in humans. **Amplitude** refers to the magnitude of oscillation of the circadian rhythm and reflects the robustness of circadian output.

**Fig. 1:**
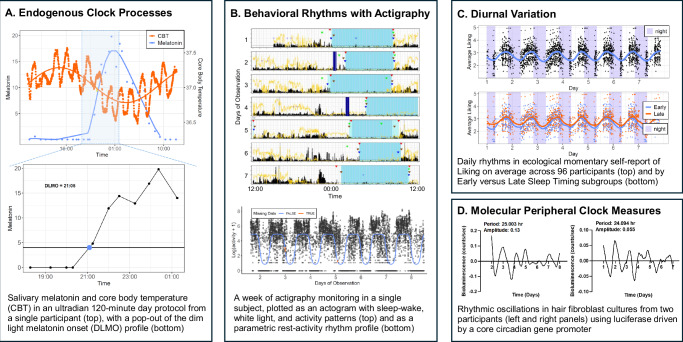
Circadian Rhythms in Psychiatry. The circadian system is a multifaceted hierarchical system that goes beyond the self-report chronotype measures used most frequently in psychiatry. **A** Endogenous clock processes captured in a 120-minute day ultradian protocol from a 15-year-old healthy female participant. A 120-minute day involves an 80-minute wake window and 40-minute sleep opportunity, which is repeated 18 times over the course of 36-hours, starting at 9:00 AM. Participants remain in semi-recumbent position in dim light (<10 lux), with isocaloric meals and time isolation in the first 24-hours. In the top panel, raw data and fitted cosinor model curves for core body temperature (CBT; orange) and raw salivary melatonin (blue) are presented across the first 24-hours of the protocol for a 15-year-old research participant. CBT, more sensitive to masking effects of sleep, dips during sleep opportunities. In the bottom panel, we depict salivary melatonin measures used to capture a gold-standard index of circadian phase, dim light melatonin onset (DLMO). DLMO can be assessed in a (relatively) shorter 6–7 h protocol, with serial saliva sampling every 30 or 60 min in dim light conditions (<10 lux). Sampling typically begins 6 h before habitual bedtime and continues 1 h after bedtime to capture the nocturnal rise in the light-sensitive hormone, melatonin. Posture, hydration, and caloric intake are commonly controlled in addition to lighting conditions. **B** A week of baseline wrist actigraphy monitoring in naturalistic conditions in the same 15-year-old healthy female participant in panel A. Wrist actigraphs leverage continuous accelerometry (and other concurrent sensor recordings, depending on the device) to capture wrist motion and estimate sleep and rest-activity rhythms. A variety of research grade and commercial accelerometry devices are in wide use. Regardless of device, raw data access is necessary for implementation of standardized open-access sleep and rest-activity rhythm analysis algorithms. The top panel is an actogram, depicting sleep (shaded light blue), environmental light (yellow line), and activity (black spikes) across 7 days (rows) plotted from noon to noon. Colored triangles indicate markers (marker press, sleep diary, light, activity) used to determine start and end of each rest interval. Navy blue shading reflects non-wear. The bottom panel is a condensed activity plot, depicting the week-long rest-activity rhythm (black lines) and best-fit parametric model (blue line) which is used to estimate markers of phase and amplitude. **C** Diurnal variation (daily rhythms) in ecological momentary assessment of subjective reward processes (self-report liking) at 6 random intervals over waking hours in 96 adolescent participants ages 13–15 yr. 24-hour rhythms in “liking” are modeled for the whole group (top panel) and by early versus late sleep timing subgroups (bottom panel). **D** Molecular measures of the peripheral clock are captured here in fibroblasts isolated from hair follicles from two adolescent research participants. Rhythmic oscillations in fibroblasts are plotted as a function of bioluminescent luciferase driven by a core circadian gene promoter. Period and amplitude are reported in hours.

Measures of **endogenous circadian rhythmicity** (Fig. 1A) are comprised of physiological markers that reflect central pacemaker timing in controlled conditions, which involve uncoupling behavioral cycles from time cues and rigorous control for masking effects (e.g., light, posture, activity, etc.) for over 24-hours. Serial sampling of hormones (melatonin, cortisol) or core body temperature enable estimation of endogenous phase, amplitude, and period. Assessment of cognitive, behavioral, affective, and arousal measures in these protocols have also uncovered circadian variation in processes central to most psychiatric conditions. Circadian phase can be measured using briefer protocols, including dim light melatonin onset (DLMO) and cortisol awakening response (CAR), or proxied with computational approaches leveraging actigraphy or photometry. Repeated measures of endogenous phase have indicated that **phase shift** patterns over time (i.e, phase advances and delays) are relevant to psychiatric outcomes, such as Lithium response and relapse prediction in bipolar disorder. **Misalignment** of endogenous phase with self-report/behavioral phase is also salient to mental health.

Behavioral and self-report measures capture circadian function in the context of socio-environmental influences. Actigraphy is a flexible ambulatory assessment tool that can index **rest-activity rhythms** (i.e., timing, amplitude of activity patterns) and circadian-relevant sleep-wake patterns (i.e., sleep midpoint) (Fig. 1B). Attenuated rest-activity amplitude and later sleep midpoint are observed in multiple psychiatric conditions (e.g., psychotic, bipolar, depressive, substance use disorders). Self-report **circadian preference** and **chronotype** measures are widely used metrics in psychiatry, capturing individuals’ circadian timing preference and manifest behavior, respectively. **Diurnal patterns**, reflecting rhythmic variation across the waking day, can be captured using repeated biobehavioral measurements, ecological momentary assessment (Fig. 1C) or passive sensing.

**Gene expression rhythms** measured in peripheral tissues capture peripheral and central oscillators. For example, buccal cells collected multiple times over the light/dark cycle or blood samples collected 12 h apart can estimate phase and amplitude of peripheral oscillators. Endogenous period can be measured in fibroblast cultures using a reporter gene such as luciferase driven by a core circadian gene promoter (Fig. 1D).

## Evaluating entrainment cues

Individuals with psychiatric illness also exhibit altered photoentrainment sensitivity (e.g., higher in bipolar disorder, lower in depression) and poorer stability of exogenous zeitgebers, which destabilize circadian rhythms. Photic sensitivity is evaluated by the degree to which the dark-sensitive circadian hormone, melatonin, is suppressed by light in the evening (melatonin suppression) or the sensitivity of the ipRGC-mediated pupil response (post-illumination pupil response). Regarding exogenous zeitgebers, ambulatory photometry captures real-time light exposure and surveys like the Social Rhythm Metric evaluate non-photic zeitgeber timing.

## Considerations for psychiatry research and treatment

Circadian dysregulation is implicated in the genetic vulnerability and pathophysiology of many psychiatric conditions. Major developmental changes in circadian rhythms over adolescence coincide with the onset of major psychiatric conditions [2]. Furthermore, most momentary biobehavioral and subjective processes are regulated by the circadian clock [1], thus, considering circadian rhythms in study designs could improve psychiatric neuroscience. Finally, accelerometry has emerged as an extremely valuable passive monitoring tool to capture real-time shifts in rhythmicity predictive of clinical status changes.

Shifting circadian phase, increasing rhythm robustness, and normalizing period predict clinical response to psychiatric treatments and serve as the basis for chronotherapies [4]. For psychotropic medications, circadian features predict therapeutic effects (e.g., shorter molecular circadian period predicts response to Lithium) and targeted circadian timing of drug administration can significantly affect efficacy and side effect profile [5]. Several interventions directly manipulate exogenous zeitgebers to stabilize aspects of the circadian system, such as Bright Light Therapy, Dark Therapy, Triple Chronotherapy, or Interpersonal and Social Rhythm Therapy (see **Glossary**). Circadian rhythm sleep-wake disorders are highly comorbid with psychiatric conditions and can be treated with adjunctive pharmacologic (melatonin, melatonin receptor agonists), chronotherapeutic, or behavioral approaches (e.g., transdiagnostic sleep-circadian intervention). While melatonin is commonly prescribed for neurodevelopmental and other psychiatric disorders, dosing standards are still being developed, and providers should be mindful about dosage/content in over-the-counter formulations (see **Glossary**). Gaining familiarity with signs of circadian dysregulation can open opportunities to deliver acceptable, low-cost circadian interventions that enhance clinical outcomes.

**Table:** Glossary Circadian features and interventions relevant to mental health research.

Feature	Definition	Assessments	Clinical Relevance
Endogenous Circadian Rhythm	Approximately 24-hour rhythms generated by the master pacemaker, the suprachiasmatic nucleus of the hypothalamus, entrained by external time cues.	Melatonin, Cortisol, Core Body Temperature measured in the context of constant routine or forced desynchrony protocols that allow circadian variation and sleep-wake dependency to be disentangled. Molecular rhythms in fibroblasts assess endogenous circadian period.	Delayed phase in ADHD, Depression; Lower cortisol awakening response in depression; low amplitude melatonin rhythm in autism spectrum disorders
Rest-Activity Rhythms	Rhythms in 24-hour activity patterns that proxy circadian rhythms in a free-living setting, using actigraphy	Actigraphy (wrist, ankle, torso, arm) or accelerometry using passive monitoring methods	Lower rest-activity rhythm amplitude in bipolar disorder, psychosis, and depression; phase advance during mania and delay during depression in bipolar disorder
Diurnal Pattern	Predictable fluctuation of mood, energy, or other psychiatric symptoms within a 24-hour period	Any measure repeated over the course of the day	Diurnal variation in mood in depression
Circadian Misalignment	When the endogenous circadian rhythm is out of sync with the external environment (light/dark cycles) or behavior (eating/sleeping times)	Deviation between an endogenous circadian rhythm measure and a self-report or behavioral measure	Greater circadian misalignment is associated with alcohol use
Circadian Preference	Individuals’ preference to sleep or be more active/alert at certain times of day, rated on a scale of morningness to eveningness	Morningness Eveningness Questionnaire, Composite Scale of Morningness	Eveningness preference is observed in most psychiatric conditions
Chronotype	Individuals’ natural tendency to sleep or be more alert at certain times of day, usually captured through behavioral sleep patterns ranging from early (morning lark) to late (night owl).	Munich Chronotype Questionnaire Midsleep on Free Days (Adjusted for Sleep Duration)	Later chronotype is observed in most psychiatric conditions
**Intervention**	**Description**		
Exogenous Melatonin	Exogenous melatonin can help shift circadian phase and treat sleep onset difficulties; longer-acting formulations may also treat sleep maintenance difficulties. Optimal melatonin dosing varies by age, diagnosis, and therapeutic goal; international guidelines and knowledgeable healthcare providers should be consulted. Nations vary in their classification of melatonin as an over the counter or prescription-only product. For over-the-counter supplements, it is advisable to select a formulation that meets national quality standards (e.g., U.S. Pharmacopeia [USP] in the United States) to ensure reliable melatonin content.		
Bright Light Therapy	A non-invasive treatment involving daily, 30–60-minute sessions of bright light (10,000 lux broad spectrum white or lower lux levels of blue-enhanced light), typically in the morning upon waking. Bright light therapy improves seasonal and non-seasonal depression, cognition, sleep, circadian rhythm sleep-wake disorders and daytime alertness.		
Dark Therapy	A behavioral intervention involving prolonged exposure to darkness during the evening/biological night (e.g., 6 PM to 8 AM) or wearing blue light-blocking glasses for 2–3 h before bedtime.		
Triple Chronotherapy	Leverages sequential sleep deprivation, circadian phase advance, and bright light therapy over the course of several days to a week to rapidly reset the sleep-circadian regulatory system and produce a relatively rapid therapeutic effect on depression or suicidal behavior.		
Interpersonal and Social Rhythm Therapy (IPSRT)	Social rhythm therapy focused on reinforcing the stability of exogenous zeitgebers, including the timing of sleep, meals, activity, and social interactions, to improve the robustness of circadian function. IPSRT is an evidence based psychosocial treatment for bipolar disorder and depression.		
